# Involvement of *LeMDR*, an ATP-binding cassette protein gene, in shikonin transport and biosynthesis in *Lithospermum erythrorhizon*

**DOI:** 10.1186/s12870-017-1148-6

**Published:** 2017-11-13

**Authors:** Yu Zhu, Gui-Hua Lu, Zhuo-Wu Bian, Feng-Yao Wu, Yan-Jun Pang, Xiao-Ming Wang, Rong-Wu Yang, Cheng-Yi Tang, Jin-Liang Qi, Yong-Hua Yang

**Affiliations:** 1 Institute of Plant Molecular Biology, State Key Laboratory of Pharmaceutical Biotechnology, School of Life Sciences, Nanjing University, No. 163 Xianlin Avenue, Qixia District, Nanjing, 210023 People’s Republic of China; 2 Co-Innovation Center for Sustainable Forestry in Southern China, Nanjing Forestry University, Nanjing, 210037 People’s Republic of China

**Keywords:** ABC transporter, *LeMDR*, Hairy root, Overexpression, RNAi, Shikonin

## Abstract

**Background:**

Shikonin is a naphthoquinone secondary metabolite with important medicinal value and is found in *Lithospermum erythrorhizon*. Considering the limited knowledge on the membrane transport mechanism of shikonin, this study investigated such molecular mechanism.

**Results:**

We successfully isolated an ATP-binding cassette protein gene, *LeMDR*, from *L. erythrorhizon*. *LeMDR* is predominantly expressed in *L. erythrorhizon* roots, where shikonin accumulated. Functional analysis of *LeMDR* by using the yeast cell expression system revealed that LeMDR is possibly involved in the shikonin efflux transport. The accumulation of shikonin is lower in yeast cells transformed with *LeMDR*-overexpressing vector than that with empty vector. The transgenic hairy roots of *L. erythrorhizon* overexpressing *LeMDR* (MDRO) significantly enhanced shikonin production, whereas the RNA interference of *LeMDR* (MDRi) displayed a reverse trend. Moreover, the mRNA expression level of *LeMDR* was up-regulated by treatment with shikonin and shikonin-positive regulators, methyl jasmonate and indole-3-acetic acid. There might be a relationship of mutual regulation between the expression level of *LeMDR* and shikonin biosynthesis.

**Conclusions:**

Our findings demonstrated the important role of *LeMDR* in transmembrane transport and biosynthesis of shikonin.

**Electronic supplementary material:**

The online version of this article (10.1186/s12870-017-1148-6) contains supplementary material, which is available to authorized users.

## Background

Shikonin and its derivatives, which are naphthoquinone pigments synthesized in the roots of the medicinal plant *Lithospermum erythrorhizon*, possess multiple medicinal and pharmacological properties, such as antibacteria, anti-inflammatory, and antioxidant [1–3]. Extensive research has also confirmed the antitumor properties of shikonin and its derivatives [4]. However, the natural resources of *L. erythrorhizon* have become scarce. In this regard, the two-stage culture system of callus cell culture and hairy roots of *L. erythrorhizon* has been established as an efficient method for producing useful compounds; in this system, (i) the callus cells or hairy roots are first cultured in a B5 growth medium for rapid proliferation and (ii) transferred into a M9 production medium to efficiently induce the biosynthesis of shikonin and its derivatives [5–8]. This system also exhibit potential for elucidating the molecular mechanisms of shikonin biosynthesis.

The metabolic pathway of shikonin biosynthesis has been intensively studied. After their biosynthesis in the endoplasmic reticulum, shikonin and its derivatives are postulated to be compartmented in red granules localized in the apoplastic space of cells [6]. The synthesized shikonin and its derivatives are then transported to epidermal cells through a method similar to transport of wax, a lipophilic compound [9]. However, a study reported that the ATP-binding cassette (ABC) transporter (AtWBC12/CER5) also plays an important role in the transmembrane transport of lipophilic compound wax [10]; the *Arabidopsis CER5* gene encodes an ABC transporter localized in the plasma membrane of epidermal cells and exports lipid metabolites to the cuticle. Hence, ABC transporters could be involved in transport of shikonin and its derivatives, which are also lipophilic compounds.

The ABC transporter superfamily is one of the largest transporter protein families in plants [11, 12]. Plant ABC transporters possess diverse transport substrates, including lipids, auxin, fatty acids, xenobiotics, heavy metals, and secondary metabolites [13]. Although the transport mechanism of shikonin metabolites remains unknown, several different alkaloid transporters have been reported. The CjMDR transporter is involved in translocation of berberine from the root to the rhizome by transporting it in the plasma membrane of cells around the xylem of the rhizome [14]. The multidrug-resistance protein (MDR), which belongs to the ABCB subfamily, is involved in transport of many divergent compounds [15]. The Nt-JAT transporter unloads nicotine secondary metabolites from the aerial part of a plant to the vacuoles [16]. Hence, other secondary metabolite transporters must be identified.

In our previous transcriptome study, we identified the ABC transporter gene *LeMDR*, which was significantly up-regulated in cells cultured in M9 production medium compared with that in B5 growth medium. We speculate that the ABC transporter gene of *L. erythrorhizon* plays an important role in transport of shikonin and its derivatives. To investigate their actual roles in shikonin transport, we cloned the full-length cDNA of *LeMDR* and analyzed its expression patterns. The function of *LeMDR* in the transport of shikonin was also investigated using the yeast mutant heterologous expression system. We also analyzed the occurrence of mutual regulation between the expression of *LeMDR* and shikonin production via overexpression (OE) and RNA interference (RNAi) of *LeMDR* in the hairy root system of *L. erythrorhizon*. This study provides new insights to elucidate the transport and biosynthesis regulatory mechanisms of shikonin and its derivatives.

## Methods

### Plant materials and growth conditions

*L. erythrorhizon* seeds were stratified in humid sands at 4 °C for approximately 4 weeks. The germinated seeds were grown on soil in growth chambers with 100 μmol·m^−2^·s^−1^ light in a 16-h light/8-h dark cycle at 25 °C. For growth under sterile conditions, the seeds were sterilized and grown in culture on half-strength Murashige and Skoog (MS) medium [17]. Ten-month-old *L. erythrorhizon* seedlings were used to analyze the tissue-specific expression of *LeMDR*.

### Molecular cloning of *LeMDR* cDNA and bioinformatics analysis

For cloning the full-length cDNA of *LeMDR*, the rapid amplification of cDNA ends (RACE) strategy was applied using SMART RACE cDNA amplification kit (Clontech, Mountain View, CA, USA). Cell cultures of *L. erythrorhizon* were harvested for RNA extraction as previously described [18]. First-strand cDNA synthesis was performed according to the manufacturer’s instructions. Gene-specific 5′ and 3′ RACE primers were designed based on the cloned sequence to obtain the full-length fragment (Additional file 1: Table S1).

Clustal W alignments of DNA and protein sequences were conducted with Megalign package (DNAStar, Madison, WI). Protein distance matrix, bootstrap values (1000 replicates), and neighbor-joining consensus trees were calculated using PHYLIP [19]. *Arabidopsis thaliana* and *Coptis japonica* sequences were recovered according to the methods proposed by Jasinski et al. [20] and Shitan et al. [21]. The GenBank accession nos. Are as follows: AtMDR1 (AAD31576.1), AtMDR2 (CAB79451.1), AtMDR3 (CAB80675.1), AtMDR4 (AAC34225.1), AtMDR5 (CAB80676.1), AtMDR6 (AAC27839.1), AtMDR7 (BAB10822.1), AtMDR8 (AAG10628.1), AtMDR9 (CAB78807.1), AtMDR10 (AAF17668.1), AtMDR11 (BAB02129.1), AtMDR12 (AAG51476.1), AtMDR13 (BAB02627.1), AtMDR14 (CAB75766.1), AtMDR15 (AAG51482.1), AtMDR16 (AAG10627.1), AtMDR17 (CAB71875.1), AtMDR18 (BAB02852.1), AtMDR19 (BAB02854.1), AtMDR20 (BAB02855.1), AtMDR21 (BAB02858.1), AtMDR22 (BAB02613.1), CjMDR (BAB62040.1).

### Genomic southern blot analysis

Southern blot analysis was performed to determine the copy number of the *LeMDR* gene in the *L. erythrorhizon* genome. The genomic DNA from the *L. erythrorhizon* seedling was extracted by using plant genomic DNA extraction kit (TaKaRa Biotech, Japan), and 5 μg of DNA was digested with *EcoR*I or *EcoR*V which not only are higher active restriction enzyme but also have no enzymatic sites in the probe sequence of *LeMDR* for Southern blot. Digested samples were separated on 0.7% agarose gel and transferred in 20 × standard saline citrate (SSC) to a charged nylon membrane (Roche Applied Science, Indianapolis, USA). The membrane was hybridized with a DNA probe encompassing the full-length *LeMDR* sequences and labeled with DIG-11-dUTP prepared by PCR. Blots were washed 2 × 5 min in 2 × SSC/0.1% SDS at room temperature, then washed 2 × 15 min in 1 × SSC/0.1% SDS at 65 °C and exposed to X-ray film.

### Expression analysis of *LeMDR*

The total RNA from the root, stem, leaf, flower, and rhizome of the ten-month-old intact of *L. erythrorhizon* was extracted by using TRIzol reagent (Takara Biotech, Japan), and 1 μg of RNA was used to synthesized cDNA by using M-MLV reverse transcriptase (Promega, USA). Real-time PCR was performed with a SYBR Green PCR Master Mix (Toyobo, Japan). The three independent RNA isolates were used for cDNA synthesis, and each cDNA sample was subjected to real-time PCR analysis in triplicate. The glyceraldehyde-3-phosphate dehydrogenase encoding gene (*GAPDH*), which has been confirmed as a best housekeeping gene in our previous reports [7, 8, 22] was used as a standard and the relative transcript levels were calculated by double delta Ct (ΔΔCt) method.

The EV hairy roots of *L. erythrorhizon* treated with DMSO (CK), 100 μM shikonin, 20 μM methyl jasmonate (MeJA), and 10 μM indole-3-acetic acid (IAA) for 3 or 8 h were extracted for *LeMDR* expression pattern analysis. The expression levels of *LeMDR* were analyzed with CFX manager software (Bio-Rad).

### Overexpression, construction of RNAi vectors, and hairy root induction

cDNAs obtained from *L. erythrorhizon* cells were used directly as PCR template for cloning the full-length cDNA of *LeMDR* with specific primers [23]. The open reading frame (ORF) of *LeMDR* was cloned into pBI121 vector [24]. The cassette containing the *Cauliflower mosaic virus* (CaMV) 35S promoter and LeMDR-eGFP (enhanced green fluorescent protein) was inserted into vectors and a pBI121-LeMDR overexpression (MDRO) construct was obtained.

A specific 390-bp sequence of *LeMDR* was used for the construction of the *LeMDR* RNA interference (MDRi) vector. A fragment was generated from *L. erythrothizon* cDNA by using the PrimeSTAR Max DNA Polymerase (Takara) and specific primers with *Bam*HI and *Xba*I restriction sites. The resulting PCR product was subcloned into a PCR-blunt vector and subsequently inserted in sense orientation downstream of the GA20 oxidase intron in the pUCC-RNAi vector as described by Chen et al. [25]. The same fragment using specific primers with *Spe*I and *Bgl*II restriction sites was ligated in antisense orientation of pUCC-RNAi already carrying the sense fragment. Finally, the resulting RNAi fragment was excised from pUCC-RNAi using the flanking *Spe*I/*Xba*I sites and inserted into the *Xba*I site of pBI121 vector.

Genetic transformation of *Agrobacterium rhizogenes* strain ATCC15834 harboring the pBI121-*eGFP* (EV), pBI121-*LeMDR*-Overexpression (MDRO), or pBI121-*LeMDR*-RNAi (MDRi) plasmid were used as infection strains for hairy roots induction. The explants of *L. erythrorhizon*, including the root, stem, and leaf of sterilized seedling (1 to 1.5 cm), were cut off. The explants were placed on 1/2 MS medium containing 0.2 mg/L 1-naphthaleneacetic acid, 2.0 mg/L 6-benzylaminopurine, and 10 μM acetosyringone and incubated for 1 or 2 days at 25 °C in the dark. Then, the explants were infected with ATCC15834. After 2 to 3 weeks of cultivation, some hairy roots appear.

### Subcellular localization of LeMDR in hairy roots and onion epidermis cells

For stable expression, the transgenic hairy roots overexpressing *LeMDR* were induced as mentioned above and grown on B5 medium for 2 weeks. For transient expression in onion epidermal cells, pBI121-*LeMDR-eGFP* and pBI121-*eGFP* were transformed into *Agrobacterium* strain GV3101. Onion epidermis cell layers were transfected with the constructs pBI121–*LeMDR–eGFP* and pBI121-*eGFP* after coating with 0.6-μm gold microparticles using a particle inflow gun as described in Ibrahim et al. [26]. After bombardment at low-pressure helium flow (26 psi), epidermal layers were incubated at room temperature for 16 to 24 h in the dark.

For the detection of eGFP localization, conventional fluorescence microscopy using an Olympus IX-70 microscope with *λ*_ex_ = 488 nm and *λ*_em_ = 510 nm was used to screen the hairy roots and onion epidermis cells with eGFP signals.

### Functional analysis of *LeMDR* in yeast cells

*LeMDR* cDNA (3.9 kb) was subcloned into the yeast expression vector pDR196 [27]. The resulting plasmid, pDR-LeMDR, was used to transform the yeast ABC mutant strain *AD12345678* (*yor1*∆, *snq2*∆, *pdr5*∆, *pdr10*∆, *pdr11*∆, *ycf1*∆, *pdr3*∆, *pdr15*∆) [28]. The yeast transformant was precultured in 50 mL of SD medium (−uracil), harvested at *A*_600_ = 1.0, and suspended by a 50-mL half-strength SD medium (−uracil) containing 1 mM shikonin, which was dissolved in 0.1% DMSO. The cells were incubated at 30 °C with shaking at 180 rpm, harvested at the indicated times by centrifugation, and washed three times with sterile Milli-Q water [16]. Next, yeast cells were disrupted with acid-washed glass beads in methanol. The samples were centrifuged and supernatants were filtered for high performance liquid chromatography (HPLC) analysis [29].

### Measurement of shikonin content in the hairy roots

The MDRO, MDRi, and control (EV) hairy roots were maintained in a growth medium (B5) [30] for 15 days with shaking at 80 rpm at 25 °C. Then, the hairy roots were transferred into M9 production medium [5], maintained on a rotary shaker at 80 rpm, and grown at 25 °C in the dark for 6 days. Both fresh hairy roots and the M9 production medium were extracted with methanol, and the extracts were analyzed by HPLC [29].

### Statistical analysis

Statistical analyses were performed using the SPSS 17.0 software (IBM, IL, USA). Student’s t-test was used for comparison between the groups. Error bars indicate the standard deviation for three biological replicates and *P* < 0.05 (*) or *P* < 0.01 (**) was considered as statistically significant.

## Results

### Cloning and sequence analysis of *LeMDR*

**Fig. 1:**
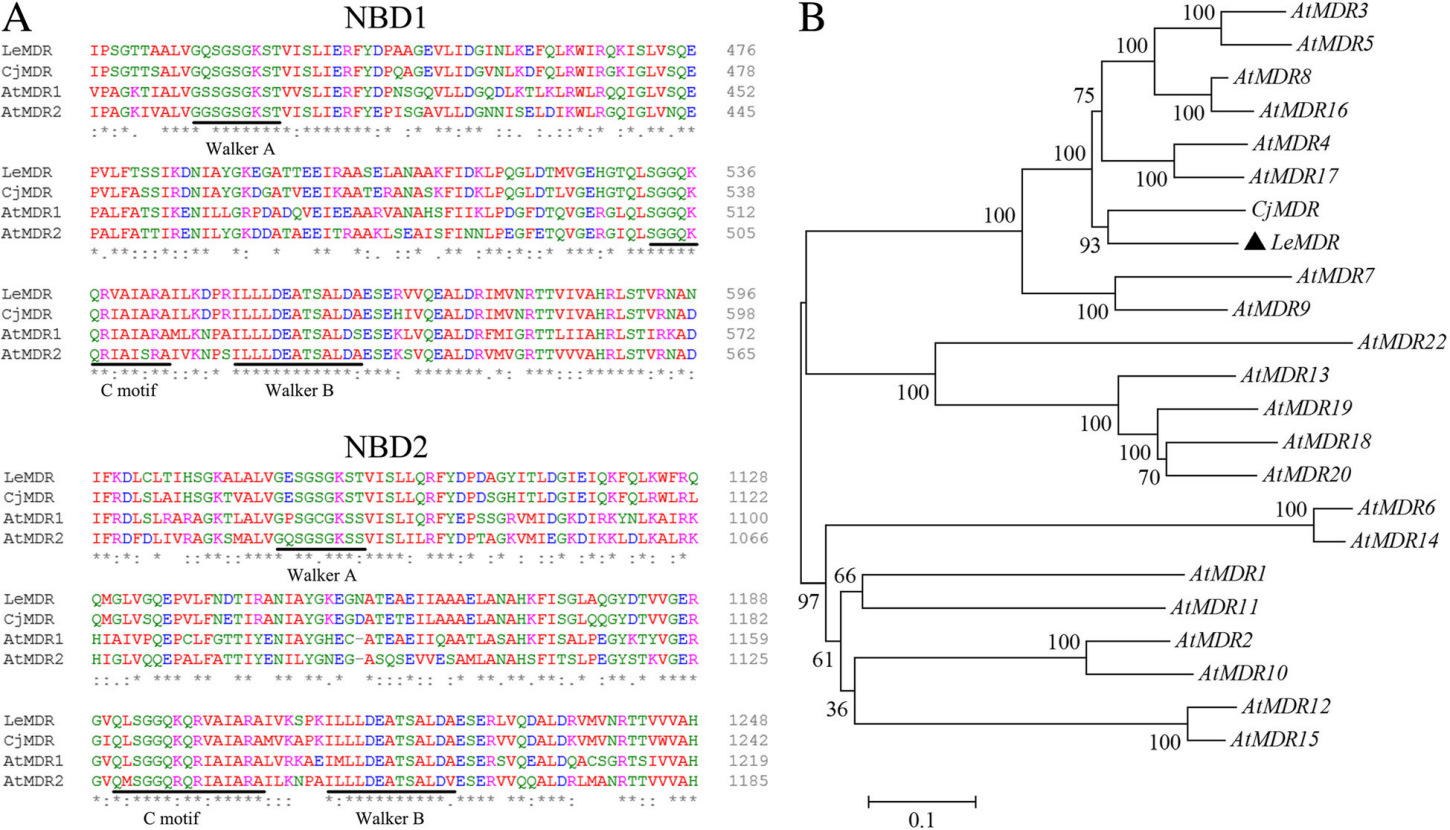
Molecular characterizations of *LeMDR*. **a** ABC motifs in the predicted LeMDR. Alignment of the NBD of LeMDR, *A. thaliana* MDR1 and MDR2, and *C. japonica* MDR showing the Walker A and B box and the ABC signature motifs. **b** Comparative phylogenetic analysis of MDRs from *A. thaliana, C. japonica* and *L. erythrorhizon*

Following differential expression profiling analysis of the transcriptomes between the cells cultured in B5 and M9 media, we discovered a transcript (isotig02082) which was significantly up-regulated in M9 medium (Additional file 2: Figure S1A). We then isolated its full-length cDNA. Blastx analysis indicated that this gene is an ABCB member of the ABC transporter family. In the phylogenetic relationship of plant ABCB transporter, it belonged to the MDR subfamily and was designated as *LeMDR* (GenBank accession number: KY293693). We then designed the gene-specific primers (Additional file 1: Table S1) and cloned its full-length ORF (Additional file 2: Figure S1B). *LeMDR* has a typical domain structure containing two nucleotide-binding folds (NBF1 and NBF2). The NBF region contains the highly conserved Walker A and B motifs as well as a sequence known as the ABC signature [15] (Fig. 1a). *LeMDR* is 3.9 kb in length and encodes putative polypeptides composed of 1296 amino acids and 9 putative transmembrane domains (Additional file 3: Figure S2). *LeMDR* is most closely related to *Coptic japonica CjMDR* (Fig. 1b). Given the presumed role of *CjMDR* involved in the translocation of berberine [21], *LeMDR* was chosen to verify our assumption that LeMDR protein was possibly involved in the transport of secondary metabolite of *L. erythrorhizon.*

**Fig. 2:**
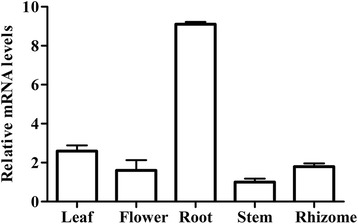
Transcript level of *LeMDR* in the root, stem, leaf, flower, and rhizome tissues of intact *L. erythrorhizon* seedlings, and the house-keeping gene *GAPDH* was used as reference. Error bars indicate the standard deviation for three biological replicates

The tissue-specific expression analysis in root, stem, leaf, flower, and rhizome suggested that the transcripts of *LeMDR* dominantly expressed in the root (Fig. 2), where shikonin and its derivatives were synthesized. The genomic southern blot analysis result showed that only one band appeared in each lane in the hybridizations (Additional file 4: Figure S3), indicating that *LeMDR* genes possibly exist as a single copy in the *L. erythrorhizon* genome.

### LeMDR is localized in the plasma membrane

**Fig. 3:**
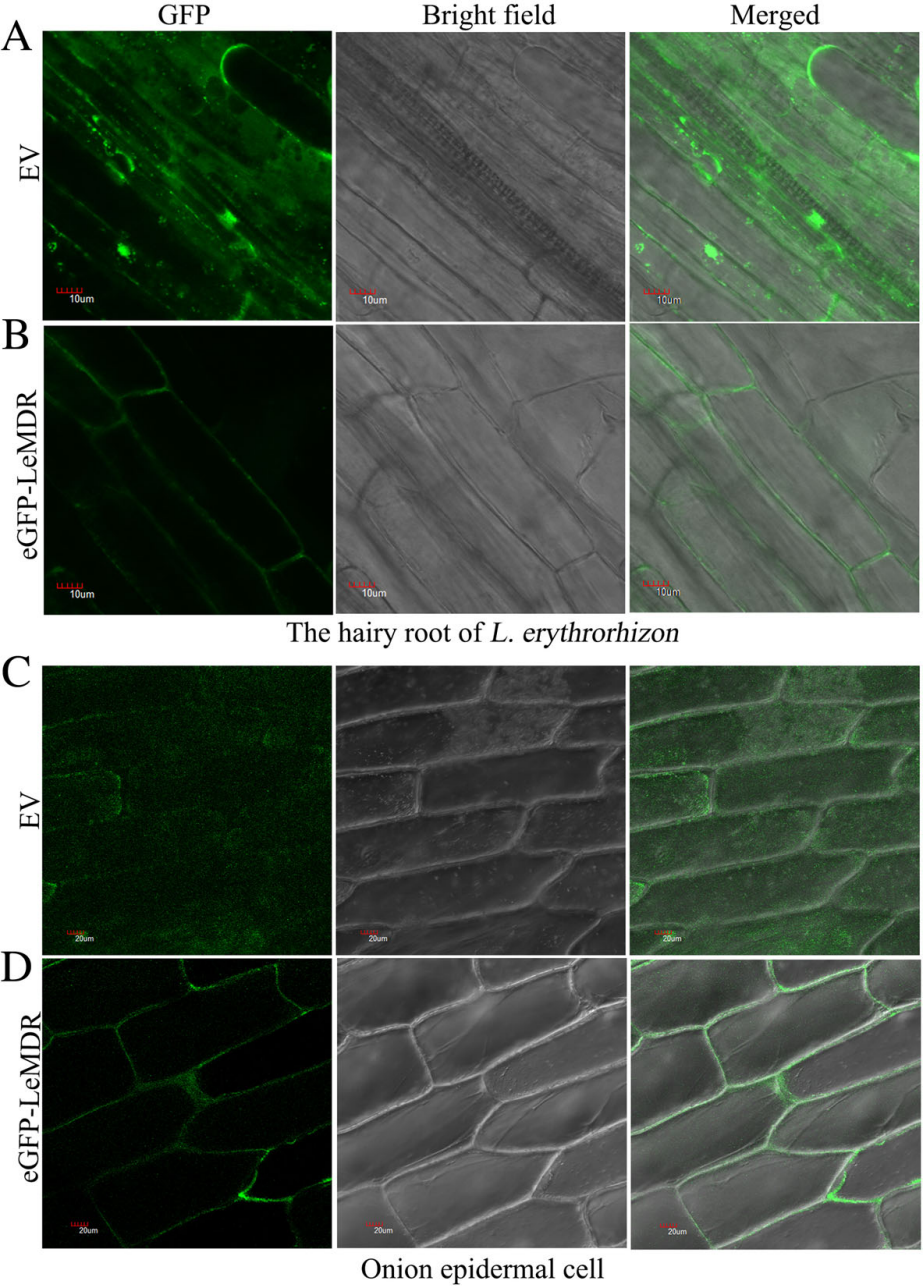
Subcellular localization of *LeMDR*-eGFP fusion protein. **a**–**b** Localization of eGFP (EV) and *LeMDR*-eGFP to plasma membranes in the hairy roots of *L. erythrorhizon*. **c**–**d** Localization of eGFP and *LeMDR*-eGFP to plasma membranes in onion epidermal cells

To investigate the cellular location of LeMDR, the full-length *LeMDR* cDNA was C-terminally fused to eGFP and expressed under the control of the CaMV 35S promoter in a fusion cassette. We respectively expressed *LeMDR-eGFP* in the hairy roots and the onion epidermis cells. Confocal microscope analysis of hairy roots (Fig. 3a–b) and heterozygous transgenic onion epidermis cells (Fig. 3c–d) showed green fluorescence signals were predominantly visible in the plasma membrane, whereas epidermal cell transformed with vector control revealed that green fluorescence signals were distributed throughout the cells. Expression of this gene in hairy roots and in onion epidermal cells suggested its plasma membrane localization.

### LeMDR functions as a shikonin transporter

**Fig. 4:**
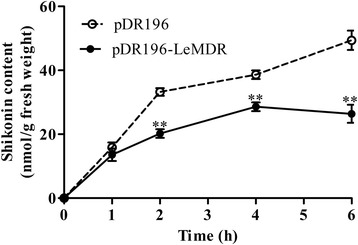
LeMDR-mediated shikonin transport in yeast cells. Time course analysis of shikonin transport in *LeMDR*-expressing yeast cells. EV control (pDR196) and *LeMDR-*expressing (pDR196–LeMDR) yeast cells were incubated in half-strength synthetic dropout medium supplemented with 1.0 mM shikonin. Shikonin was analyzed by HPLC. The error bars represent standard deviations from three biological replicates. Asterisks indicate statistically significant difference compared with control EV (pDR196) (** *P* < 0.01)

To examine the function of LeMDR as a shikonin transporter, *LeMDR* was expressed with a shuttle vector, pDR196, with which a foreign gene is constitutively expressed by PMA1 promoter [27], in *Saccharomyces cerevisiae* mutant strain *AD12345678*. The strain *AD12345678* lacks eight major yeast ABC transporter-encoding genes that confer MDR [28]. The same yeast strain transformed with the EV (pDR196) was used as a negative control. Given that LeMDR was suggested to possess export transport activity for shikonin, the time course of shikonin uptake was monitored quantitatively by HPLC analysis. The result showed that the shikonin level in LeMDR-expressing yeast cells is dramatically lower than that in EV yeast cells (Fig. 4).

Moreover, we also investigated the substrate specificity by estimating drug sensitivity in yeast transformants. A significant difference in drug sensitivity between LeMDR transformant and the control was found between the 5,8-dihydroxy-naphthoquinone (DNQ) and shikonin, the side chain of the latter cyclized to give an unusual four-membered ring system. The data suggested that shikonin was recognized as a substrate of LeMDR and exported from the *LeMDR*-expressing yeast vesicles (Additional file 5: Figure S4). However, DNQ accumulated in similar levels as in pDR196-expressing vesicles, indicating that shikonin had relative substrate specificity.

### *LeMDR* affects shikonin biosynthesis in *L. erythrorhizon* hairy roots

**Fig. 5:**
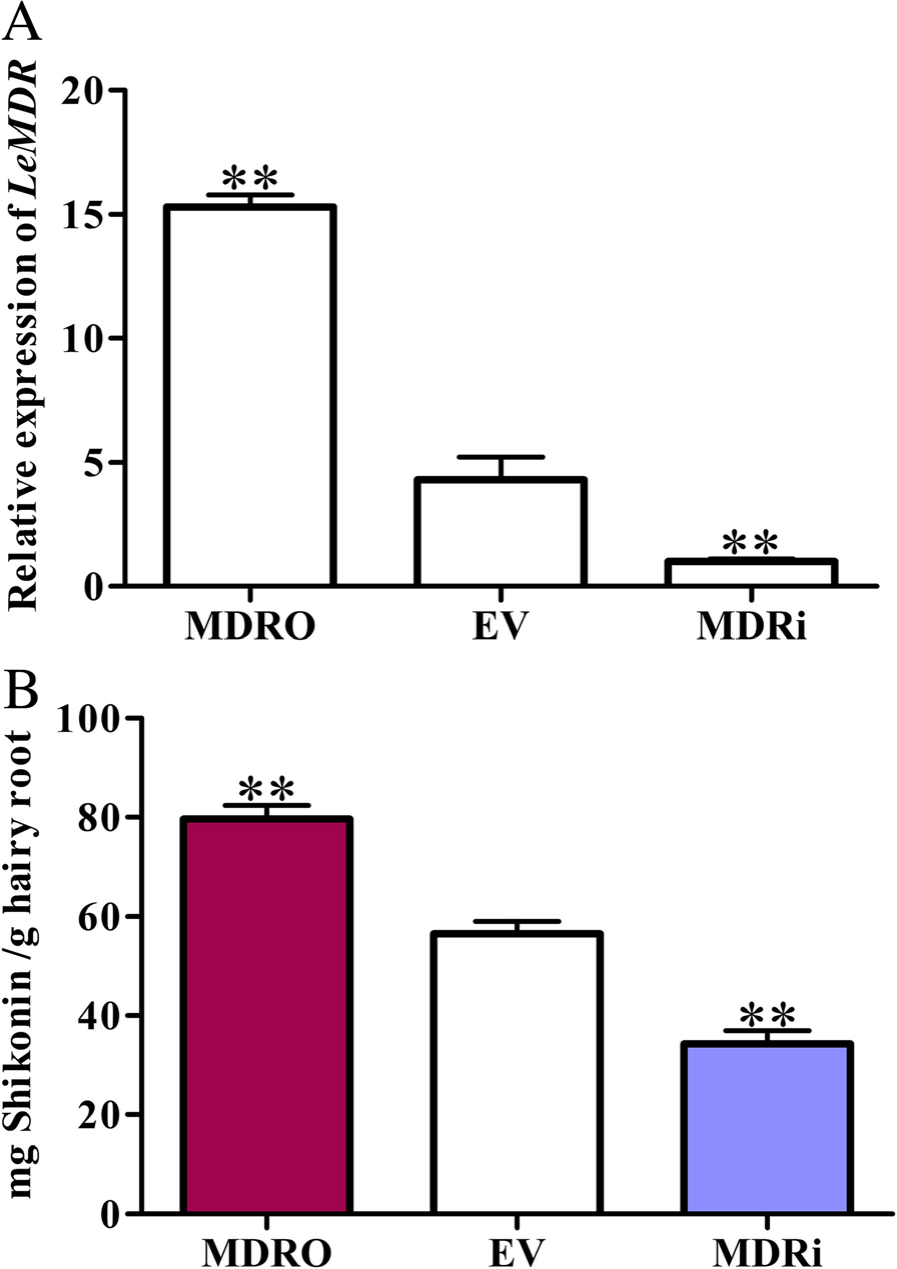
Accumulation of shikonin in the hairy roots was analyzed by HPLC. After transgenic hairy root were cultured in B5 liquid medium for 2 weeks, MDRO, EV, and MDRi hairy root lines were transferred to M9 liquid medium in the dark for 6 days. **a** Expression analysis of *LeMDR* in MDRO, EV and MDRi hairy roots by real-time PCR, and the housekeeping gene *GAPDH* was used as control. **b** HPLC analysis of shikonin content in MDRO, EV and MDRi hairy roots. The error bars represent SD from three biological replicates, and asterisks indicate statistically significant differences of transcript or shikonin levels in MDRO and MDRi compared with EV hairy roots. * *P* < 0.05, ** *P* < 0.01

Altering the expression levels of the target gene in the OE and RNAi hairy roots of *L. erythrorhizon* had been proven to be useful for identifying genes involved in shikonin biosynthesis. Therefore, the overexpression vector MDRO and the RNAi vector MDRi were constructed (Additional file 6: Figure S5A), and pBI121-eGFP empty vector EV was used as control. After infecting with *A. rhizogenes* 15,834, hairy roots of *L. erythrorhizon* appeared at the root, stem, and leaf explants (Additional file 6: Figure S5B). The expression level of *LeMDR* in hairy roots was determined through real-time PCR to confirm the transgenic effects. The results showed that the expression level of *LeMDR* in the overexpressing hairy roots increased up to four-fold, and RNAi triggered a four-fold decline of transcript levels of *LeMDR* compared with those found in EV control (Fig. 5a).

The *L. erythrorhizon* hairy roots were cultured in hormone-free B5 medium for multiplying growth under light condition. After the hairy roots were transferred to hormone-containing M9 medium in the dark at 24 °C, shikonin and its derivatives were biosynthesized and released to the medium (Additional file 6: Figure S5C), as previously described [31]. The content of shikonin and its derivatives reached the highest level at the sixth day (Fig. 4) [8]. The data of extracted shikonin content indicated that MDRO hairy roots were found to exhibit higher concentration of shikonin than that of EV control, whereas the decline was observed in MDRi hairy roots (Fig. 5b). Basing on the result, we speculated that a significantly positive linear correlation between LeMDR expression level and shikonin production occurred. These results suggested that the biosynthesis of shikonin was also indirectly affected by LeMDR, which was possibly via the efficient transport of the shikonin product out of the hairy root cells by LeMDR.

### Expression patterns of *LeMDR*

**Fig. 6:**
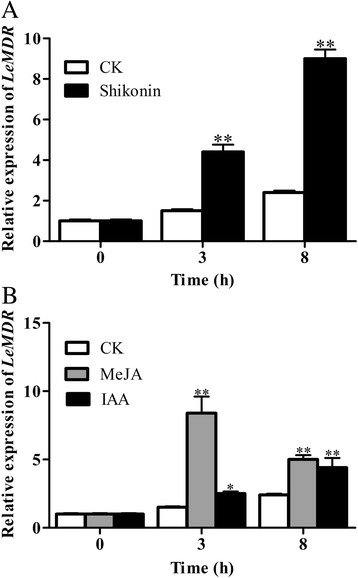
Relative expression of *LeMDR* in response to various treatments in the EV hairy roots of *L. erythrorhizon* by real-time PCR. The hairy roots of *L. erythrorhizon* were treated for 3 or 8 h with 100 μM shikonin (**a**) or 20 μM MeJA and 10 μM IAA (**b**). Results were normalized to *GAPDH* and are shown relative to the level in DMSO control hairy root (CK). The error bars represent standard deviation from three biological replicates. * *P* < 0.05, ** *P* < 0.01

To examine the occurrence of mutual regulation on the expression level of *LeMDR* and shikonin biosynthesis, we analyzed the mRNA expression level of *LeMDR* in response to the treatment of shikonin, as well as to the treatment of shikonin-positive regulators, MeJA and IAA. Treatment with IAA could significantly increase the biosynthesis of shikonin [32]. MeJA is a specific elicitor for the increased production of shikonin [33] and possesses a stimulating effect on the transcripts of key shikonin biosynthesis-related genes. Treatment of hairy roots with shikonin (Fig. 6a) and IAA (Fig. 6b) significantly increased *LeMDR* expression by four- and two-fold, respectively, within 8 h. Treatment with MeJA significantly increased the LeMDR expression up to six-fold at 3 h, and two-fold at 8 h (Fig. 6b). These results revealed that the expression of *LeMDR* was possibly involved in mutual regulation by the product concentration of shikonin.

## Discussion

ABC transporters represent one of the largest and most conserved protein families in plants [34, 35]. These proteins are ubiquitous and membrane-intrinsic transporters that utilize ATP-binding energy to drive the transport of their substrates across the biological membranes [36]. AtMDR1, the first identified plant ABC transporter protein, was cloned from *Arabidopsis thaliana* [37]. Subsequent studies have found that plant ABC transporters have diverse transport substrates including hormones [38], lipids [10], heavy metals [39], auxin [40], and xenobiotics [11]. The membrane transport of plant secondary metabolites is a relatively newly developing research area, and ABC transporters have been found to be involved in some plant systems, such as: MATE transporter of *Nicotiana tabacum*-mediated vacuolar transport of nicotine [16] and CrTPT2 transporter controls leaf surface secretion of anticancer drug components in *Catharanthus roseus* [41]. However, no ABC transporters responsible for the secondary metabolites of *L. erythrorhizon*, shikonin and its derivatives, have been reported so far.

In this study, we isolated a *LeMDR* gene from the shikonin-producing plant *L. erythrorhizon*. The bioinformatics analysis demonstrated that *LeMDR* encodes a full ABC protein and possesses the common structural characteristics of all functional MDR-type ABC transporter proteins. The domains analysis showed that *LeMDR* possessed two transmembrane domains (TMDs) and two nucleotide-binding domains (NBDs), which are arranged in the “TMD1–NBD1–TMD2–NBD2” direction. NBDs have three characteristic motifs common to the ABC transporter family, “Walker A”, “Walker B,” and C motif [42]. The protein is implied to supply the energy to transport the specific substrate by the binding and hydrolysis of ATP.

Our study showed that LeMDR is localized in the plasma membrane of *L. erythrorhizon* and expressed preferentially in the root of the intact plant where shikonin is synthesized and accumulated [43]. So, the accumulation of shikonin in the roots of *L. erythrorhizon* cultured in natural soil environment would also be beneficial for the plant because shikonin may protect the underground organs from attack by soil pathogens. The *L. erythrorhizon* transport system may be a model for understanding such a transport mechanism of shikonin.

MDR-type ABC transporters usually function as drug efflux pumps [21]. When the functionally active *LeMDR* gene was expressed in yeast, it behaved as an ABC efflux shikonin transporter. LeMDR mediates the transporter of shikonin to the root surface from the biosynthesis site of cells. LeMDR functions presumably actively in the secretion of these endogenous, potentially toxic compounds. Interestingly, the human MATE1 is also a polyspecific exporter that transports nicotine and other toxic compounds [44]. We assume that LeMDR was responsible for unloading of shikonin in the correct orientation because this transport activity was clearly observed in the yeast system.

Hairy root transformation provided an excellent model for the functional analysis of *LeMDR*. The results showed that *LeMDR* overexpression under the control of a 35S promoter in hairy root stable transformants strongly affected shikonin biosynthesis. Conversely, *LeMDR* RNAi decreased the concentration of shikonin in the hairy root lines. The data suggested that LeMDR transporter plays an important role in the accumulation of shikonin possibly by an indirect method, i.e., the efficient transport of shikonin out of the cells by LeMDR would protect the cells from damage and enhance its biosynthesis in turn. Recent study indicates that ABCB4 of *Arabidopsis* not only mediates efflux of auxin but is also regulated by intracellular auxin concentrations [45]. A characterization of the transport activity of ABCB4 showed that initial IAA accumulation was followed by IAA export [46], and a subsequent study confirmed the substrate-dependent switch to efflux [40]. We speculated that effect on the regulation of LeMDR by the biosynthesis efficiency based on shikonin effective efflux might occur. Given that shikonin could not be effectively transported into the extracellular space, accumulation of metabolites in the intracellular space would inhibit the growth and further production of shikonin [47]. Therefore, the cellular activity and the biosynthesis of shikonin significantly decreased.

Many studies have described successful strategies for the increase in production of secondary metabolites by elicitation techniques [48]. Elicitation is a method for the induction of secondary metabolite that involves the addition of any elicitor, such as MeJA and ethylene, to the culture media [49]. MeJA, as an important plant growth regulator, caused a rapid increase in the activities of enzymes involved in the biosynthesis of shikonin [50]. IAA could significantly increase the biosynthesis of shikonin [32]. If LeMDR confers shikonin transport, a mutual regulation would occur on the expression level of *LeMDR* by the shikonin product. To examine this postulation, we examined the mRNA expression level of *LeMDR* in response to the treatment of shikonin, as well as to the treatment of shikonin-positive regulators, MeJA and IAA. Our results showed that the expression of *LeMDR* was significantly up-regulated by exogenous shikonin. The *LeMDR* expression of hairy roots treated both with MeJA and IAA indicates that the increase of shikonin production may require more LeMDR for its transport. On the other hand, MeJA and IAA may also directly regulate the expression of LeMDR. These findings are consistent with the hypothesis that an effect of mutual regulation might occur between the expression of *LeMDR* and shikonin production.

## Conclusions

Shikonin, found in *L. erythrorhizon*, possesses important medicinal value. However, knowledge of its membrane transport mechanism still remains significantly insufficient. In this study, we cloned an ATP-binding cassette protein gene, *LeMDR*, from *L. erythrorhizon* and suggested its functions in the membrane transport of shikonin, by using the yeast mutant expression system, as well as its positive regulation on shikonin biosynthesis, by using the hairy root system by the overexpression and RNAi transgenic systems. Our results not only provided the possible theoretical explanation about the role of ABC transporter in shikonin metabolism, but also offer an effective method of increasing the production of secondary metabolites of medicinal plants by genetic engineering.

## Additional files

Additional file 1: Table S1. Primer sequences for cloning and expression analysis of the *LeMDR* gene. (DOC 40 kb)

Additional file 2: Figure S1. *LeMDR* cDNA cloning from cell cultures of *L. erythrorhizon*. (A) Differential expression of the ABC transcript isotig02082 (*LeMDR*) in the callus cells cultured in B5 and M9 media. (B) The PCR product of the *LeMDR* ORF. (JPEG 389 kb)

Additional file 3: Figure S2. Predicted transmembrane helices of LeMDR protein. Four transmembrane domains at the end terminal part. The *X*-axis represents the *LeMDR* amino acids position along the protein sequence. (JPEG 566 kb)

Additional file 4: Figure S3. Southern blot hybridization of *L. erythrorhizon* with the LeMDR specific probe. The genomic DNA of *L. erythrorhizon* was digested with various endonucleases. Line 1, positive control: pBI121-LeMDR recombinant plasmid; 2, *EcoR*I; 3, *EcoR*V; 4, *EcoR*I and *EcoR*V. (JPEG 684 kb)

Additional file 5: Figure S4. Substrates specificity analysis for LeMDR. Yeast vesicles were prepared from pDR196 or pDR196-LeMDR transformants. After 6 h incubation with shaking, substrate including the shikonin or DNQ accumulated in yeast cells was calculated. The error bars represent standard deviations from three biological replicates. Asterisks indicate statistically significant difference compared with control EV (pDR196). ** *P* < 0.01. (JPEG 420 kb)

Additional file 6: Figure S5. Induction and culture of the hairy roots of *L. erythrorhizon*. (A) Structure of the pBI121-eGFP transformation vectors. (B) Induced the hairy roots with root, stem, and leaf explants. (C) The hairy roots in the B5 liquid medium for multiplication, and the hairy roots in M9 medium for the production of shikonin and its derivatives. (JPEG 1779 kb)

## Abbreviations

ABC: ATP-binding cassette
DNQ: 5, 8-dihydroxy-naphthoquinone
eGFP: Enhanced green fluorescent protein
EV: pBI121-*eGFP* empty vector
*GAPDH*: Glyceraldehydephosphate dehydrogenase gene;
IAA: Indole-3-acetic acid
*LeMDR*: *L. erythrorhizon* MDR protein gene
MDR: Multidrug-resistance protein
MDRi: pBI121-*LeMDR*-RNAi
MDRO: pBI121-*LeMDR*-Overexpression
MeJA: Methyl jasmonate
NBD: Nucleotide binding domain
RACE: Rapid amplification of cDNA ends
RNAi: RNA interference
TMD: Transmembrane domain
