# Supplementary material for: Involvement of LeMDR, an ATP-binding cassette protein gene, in shikonin transport and biosynthesis in Lithospermum erythrorhizon
Source: BMC Plant Biol. 2017 Nov 13;17:198. doi: 10.1186/s12870-017-1148-6 (PMC5683320; doi:10.1186/s12870-017-1148-6)
Supplement: Supplementary file 1 — Primer sequences for cloning and expression analysis of the LeMDR gene. (DOC 40 kb) [file 12870_2017_1148_MOESM1_ESM.doc]

**Table S1** Primer sequences of cloning and expression of *LeMDR* gene

| **Primer types** | **Primer names** | **Sequences (5’-3’)** |
| --- | --- | --- |
| RACE | 3’ RACE F1 | GCTGGAGCAAGACTGGTTGACGATG |
|  | 3’ RACE F2 | CCAAGACGCCCTAGACCGAGTAATG |
|  | 5’ RACE R1 | TTCCTTTAGTTCGATGTCTCCACG |
|  | 5’ RACE R2 | AATGATGCCACCGTTCTGATTGAG |
| ORF cloning | MDR-pBI121-F | CATCCTAGGATGACTAAAGAAAATGGTGATCATGGTG |
|  | MDR-pBI121-R | CATAGATCTCGGGCCCGCAGTAGAAGCTGACATATGC |
| RNAi | MDR-RNAi-SF  MDR-RNAi-SR  MDR-RNAi-AF  MDR-RNAi-AR | CATGGATCCTTGGTTGGGCAAAGTGGTAGTGGGAAAT  CATTCTAGATAGAAGTAAAATTCGAGGGTCTTTCAAT  CATACTAGTTAGAAGTAAAATTCGAGGGTCTTTCAAT  CATAGATCTTTGGTTGGGCAAAGTGGTAGTGGGAAAT |
| PUCC-RNAi | Intron-F  Intron-R | CGGACCGTACTACTCTATTCGTTTCAA  GAAACGAATAGAGTAGTACGGTC |
| ATCC15834 | rolC-F | CTCCTGACATCAAACTCGTC |
|  | rolC-R | TGCTTCGAGTTATGGGTACA |
| Real-time PCR | LeMDR-Q-F | GAGGACATTCGTGGAGACATC |
|  | LeMDR-Q-R | GATTTCCCACTACCACTTTGC |
|  | GAPDH-Q-F | ACCGTCCACTCCATTACCG |
|  | GAPDH-Q-R | ATGAGGCAGCCCTTCCACC |
| Southern blot | LeMDR-SB-F  LeMDR-SB-R | CAAACGTCCCAATCCGTAG  CGTCAACCAGTCTTGCTCC |
| Expression vector | LeMDR-pDR-F  LeMDR-pDR-R | CCGGAATTCATGACTAAAGAAAATGGTGATCATGGTG  GCGTCGACCTAAGTAGAAGCTGACATATGCAATGAA |
