# Supplementary figures and images for: Involvement of LeMDR, an ATP-binding cassette protein gene, in shikonin transport and biosynthesis in Lithospermum erythrorhizon
Source: BMC Plant Biol. 2017 Nov 13;17:198. doi: 10.1186/s12870-017-1148-6 (PMC5683320; doi:10.1186/s12870-017-1148-6)

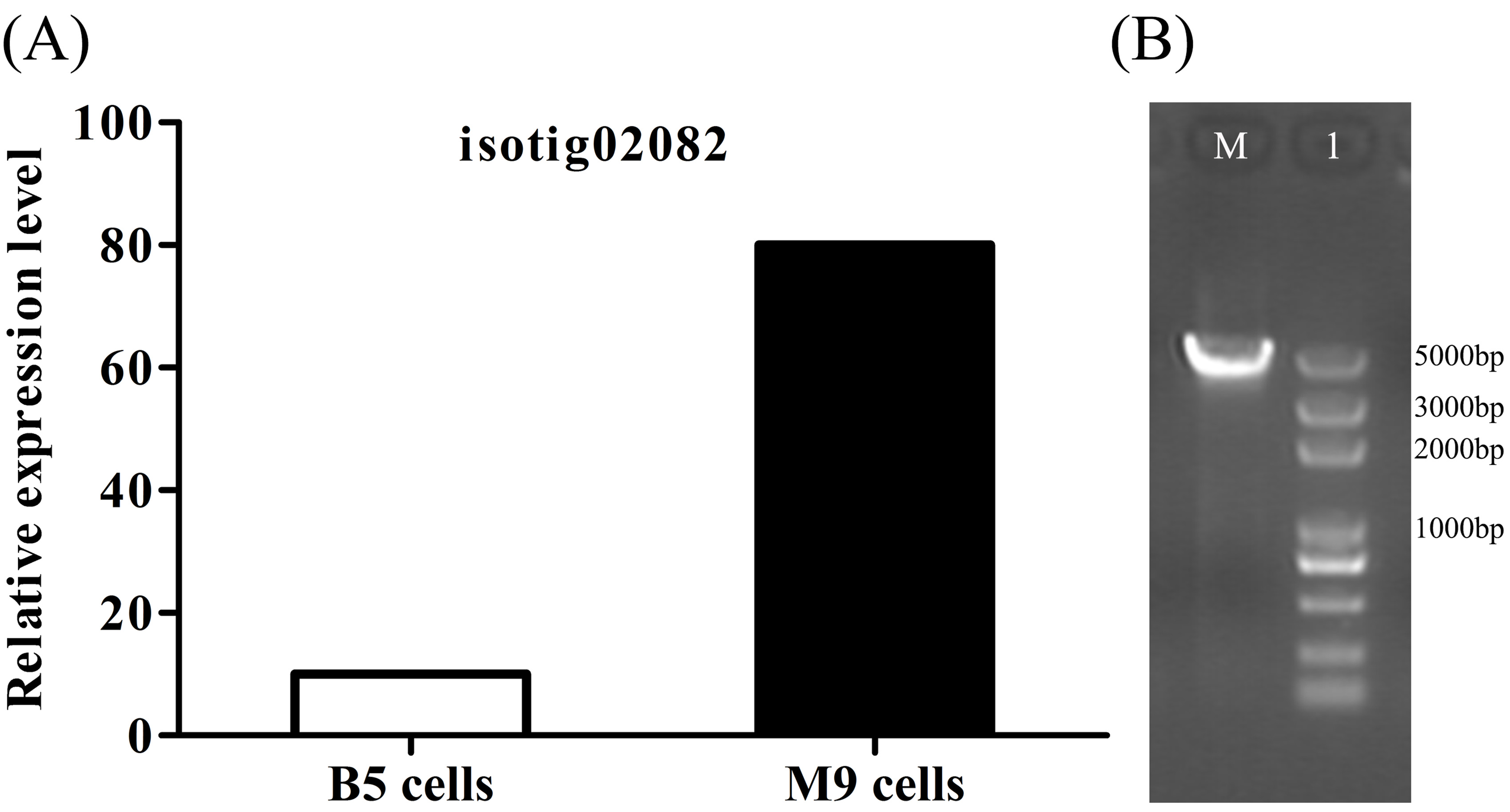

Supplement: Supplementary file 2 — LeMDR cDNA cloning from cell cultures of L. erythrorhizon. (A) Differential expression of the ABC transcript isotig02082 (LeMDR) in the callus cells cultured in B5 and M9 media. (B) The PCR product of the LeMDR ORF. (JPEG 389 kb) [file 12870_2017_1148_MOESM2_ESM.jpg]

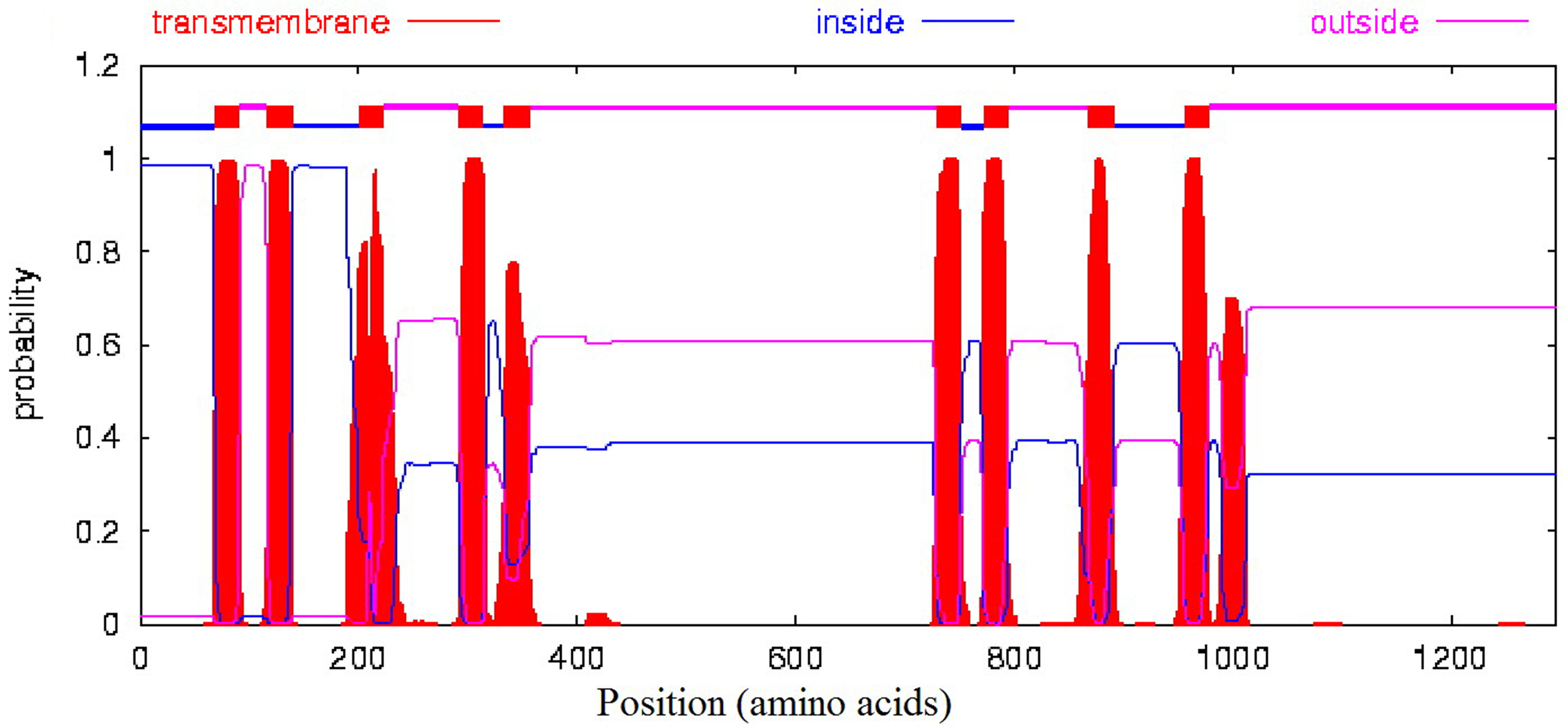

Supplement: Supplementary file 3 — Predicted transmembrane helices of LeMDR protein. Four transmembrane domains at the end terminal part. The X-axis represents the LeMDR amino acids position along the protein sequence. (JPEG 566 kb) [file 12870_2017_1148_MOESM3_ESM.jpg]

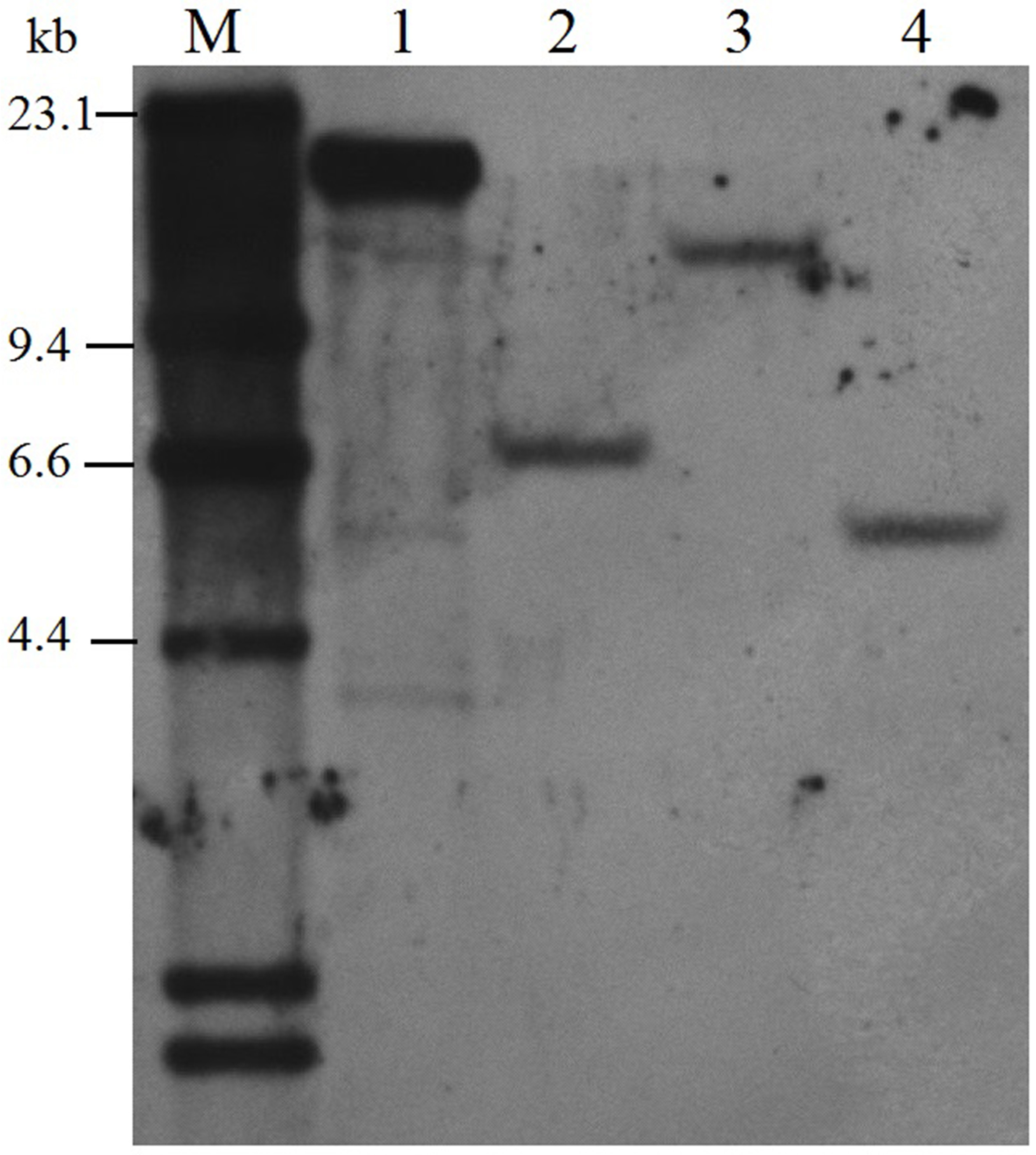

Supplement: Supplementary file 4 — Southern blot hybridization of L. erythrorhizon with the LeMDR specific probe. The genomic DNA of L. erythrorhizon was digested with various endonucleases. Line 1, positive control: pBI121-LeMDR recombinant plasmid; 2, EcoRI; 3, EcoRV; 4, EcoRI and EcoRV. (JPEG 684 kb) [file 12870_2017_1148_MOESM4_ESM.jpg]

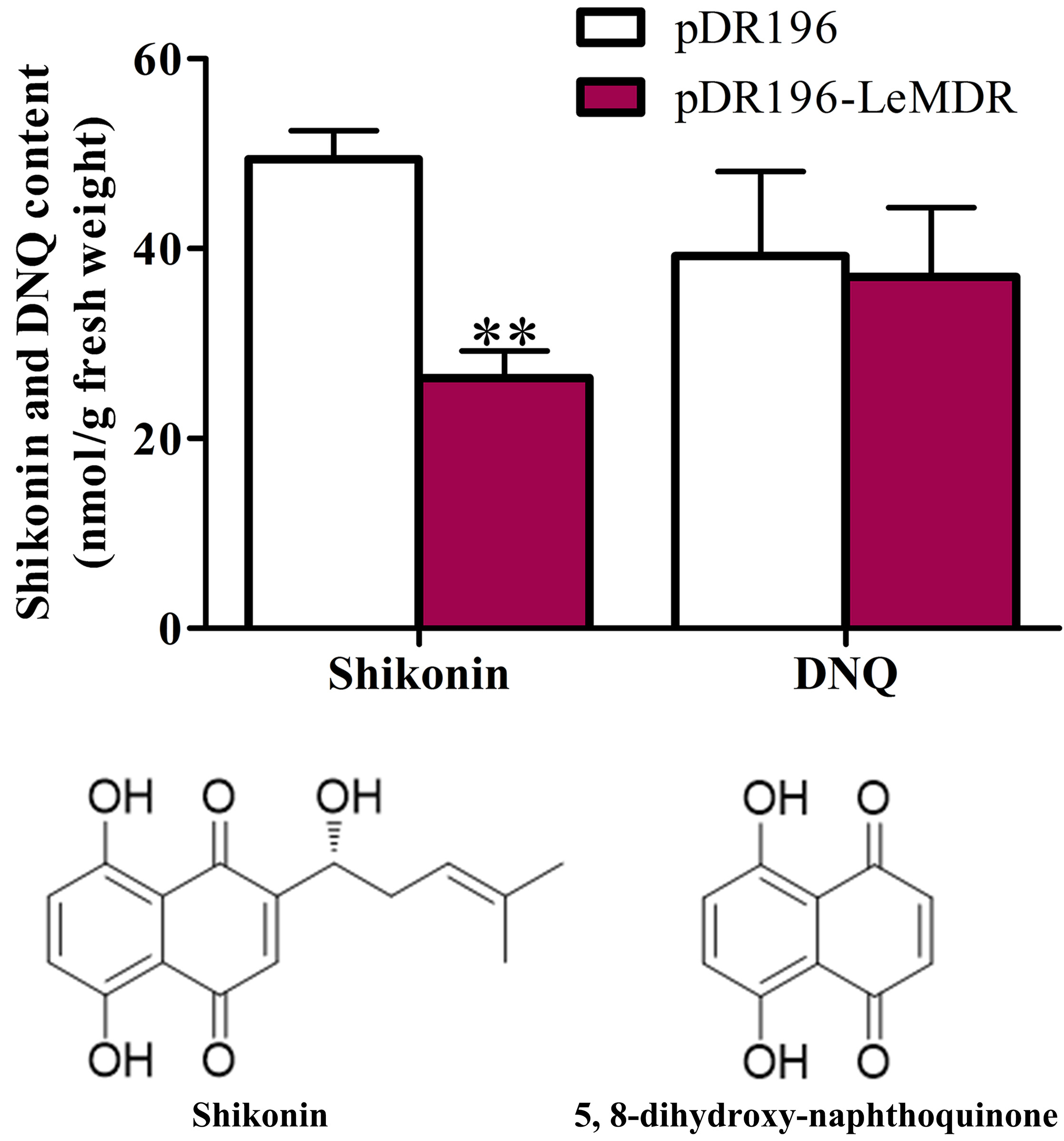

Supplement: Supplementary file 5 — Substrates specificity analysis for LeMDR. Yeast vesicles were prepared from pDR196 or pDR196-LeMDR transformants. After 6 h incubation with shaking, substrate including the shikonin or DNQ accumulated in yeast cells was calculated. The error bars represent standard deviations from three biological replicates. Asterisks indicate statistically significant difference compared with control EV (pDR196). ** P < 0.01. (JPEG 420 kb) [file 12870_2017_1148_MOESM5_ESM.jpg]

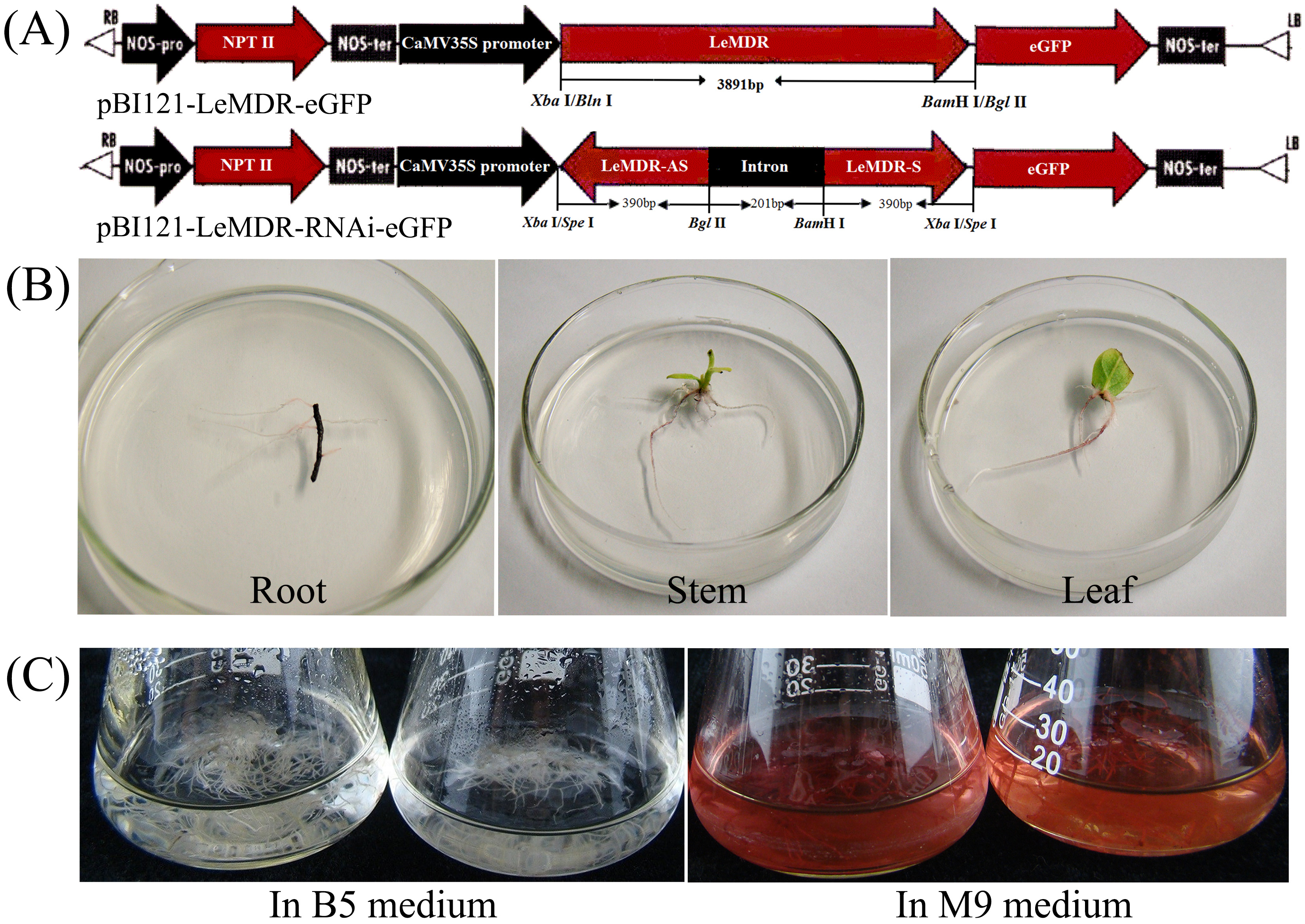

Supplement: Supplementary file 6 — Induction and culture of the hairy roots of L. erythrorhizon. (A) Structure of the pBI121-eGFP transformation vectors. (B) Induced the hairy roots with root, stem, and leaf explants. (C) The hairy roots in the B5 liquid medium for multiplication, and the hairy roots in M9 medium for the production of shikonin and its derivatives. (JPEG 1779 kb) [file 12870_2017_1148_MOESM6_ESM.jpg]
